# Cost-effectiveness Analysis of Hypoallergenic Milk Formulas for the Management of Cow’s Milk Protein Allergy in the United Kingdom

**DOI:** 10.36469/jheor.2021.26010

**Published:** 2021-08-06

**Authors:** Rui Martins, Mark P. Connolly, Eleanor Minshall

**Affiliations:** 1 Global Market Access Solutions, Health Economics Unit, St-Prex, Switzerland; 2 Unit of PharmacoEpidemiology & PharmacoEconomics, Department of Pharmacy, University of Groningen, Groningen, The Netherlands; GMAS Services LTD, London, England; 3 Sheffield Children’s NHS Foundation Trust, Sheffield, United Kingdom

**Keywords:** cost-effectiveness, atopic march, hypoallergenic formula milk, cow’s milk protein allergy

## Abstract

**Background:** Cow’s milk protein allergy (CMPA) is the most common food allergy in early childhood. In most children CMPA resolves by age 5 or 6; however, if not treated correctly can provoke nutritional deficiency resulting in poor growth. Management consists of excluding cow’s milk from the diet, with hypoallergenic formulas (or non-dairy alternatives) being introduced to meet nutritional requirements.

**Objectives:** To compare the cost-effectiveness of hypoallergenic formulas in reducing allergic manifestations and promoting immune tolerance in infants with immunoglobulin E (IgE)-mediated symptoms of CMPA.

**Methods:** A trial-based decision analytic cohort model was developed to simulate the occurrence of urticaria, eczema, asthma, rhinoconjunctivitis, or being symptom-free in infants with CMPA in the United Kingdom. Amino acid-based formula (AAF), extensively hydrolysed casein formula containing Lactobacillus rhamnosus Gorbach Goldin (EHCF+LGG), extensively hydrolysed whey formula (EHWF), and soy formula (SF) were compared using the National Health Service (NHS) perspective, 3-year time horizon and 3.5% discount rate for cost and health consequences. Hypoallergenic formulas comparative efficacy was sourced from a prospective cohort study. Resources required to manage allergic symptoms were sourced from published literature, validated by a UK clinician, and applied to UK cost resources. Results were reported as cost per additional child free from allergic manifestations at 3 years and cost per additional immune tolerant child at 3 years.

**Results:** In the base case, infants receiving EHCF+LGG were associated with lower NHS resource use and improved CMPA tolerance. Over the 3-year treatment period, savings of £119, £476, and £2645 were achieved with EHCF+LGG compared to SF, EHWF and AAF, respectively. Infant formula accounted for the largest proportion of resource consumption averaging 47% for all comparators, with a minimum of 31% for SF and a maximum of 69% for AAF over 3 years. General practitioners’ visits constituted the second highest cost component, approximately 16% of total costs across comparators. The results were robust to deterministic and probabilistic sensitivity analyses.

**Conclusions:** Compared to AAF, SF, and EHWF hypoallergenic formulas, EHCF+LGG was the most cost-effective, associated with lower total costs and contributing to a higher proportion of children being symptom-free and developing immune tolerance 3-years after diagnosis.

**Note:** An Author Correction to this article was published on October 6, 2021.

## BACKGROUND

Cow’s milk protein allergy (CMPA) is the most common food allergy in early childhood^1^ with symptoms appearing after the introduction of cow’s milk into the diet.^2^ In most children, the allergy resolves by their 5th^2^ or 6th years of age.^3^ If not treated correctly, CMPA can provoke nutritional deficiency, resulting in poor growth and decreased Vitamin D levels.^4,5^ It is also thought that CMPA occurring in infancy is the first step of an allergic march leading to a higher probability of further atopic symptoms as time progresses.^6,7^

This condition is caused by an exacerbated immune-mediated response to one or more proteins in cow’s milk. Subsequent exposure to the protein(s), elicits cross-linking of immunoglobulin E (IgE) antibodies and the release of histamines and other immune mediators from mast cells that can give rise to the immediate symptoms of urticaria and angioedema.^8,9^ CMPA is often categorized into IgE-mediated and non-IgE-mediated symptoms. IgE-mediated reactions are characterised by allergic manifestations occurring within 1 to 2 hours of ingestion of the allergen. Non-IgE symptoms manifest within hours to days.^10^ As non-IgE symptoms to cow’s milk are reminiscent of other allergic and non-allergic manifestations in infancy, delayed and erroneous diagnoses are common.^8,11,12^ Delayed diagnosis can result in faltering growth in infants^13,14^ and causes substantial anxiety for parents.^12^ Estimates of CMPA prevalence are influenced by the diagnostic criteria used, but it has been reported that 1.8% to 7.5% of infants have CMPA.^9^ In 2015, the EuroPrevall prospective birth cohort study (n=12 049 children conducted in 9 European countries) observed an overall incidence of double-blind placebo-controlled food challenge-confirmed CMPA of 0.54% in Europe.^15^ Notably, the United Kingdom had an estimated prevalence of CMPA of 1.28% at 2 years of age, this being the highest amongst those countries included in the study.^15^ IgE-mediated CMPA is thought to be more common, affecting 54% to 60% of all children with the condition compared to non-IgE-mediated CMPA (15%-45%).^8,16,17^

For non-IgE CMPA, the diagnosis is based on clinical history, symptoms, and physical examination, and confirmed by the removal and reintroduction of cow’s milk in the diet.^8,18^ CMPA allergic manifestations include urticaria, angioedema, abdominal pain, vomiting, colic, diarrhoea, blood and/or mucous in stools, constipation, or nasal congestion. Some children present with eczema that does not improve with treatment.^2,10^ Early-onset eczema resistant to treatment can also be a presentation of CMPA as can anaphylaxis (airway obstruction, breathing difficulties, pallor, drowsiness, or hypotension).^2,19^ CMPA management consists of completely excluding cow’s milk from the infant’s diet, with hypoallergenic formulas (or non-dairy alternatives) being introduced to meet nutritional requirements in non-breastfed children.^9,18^

Previous economic analyses were conducted assessing the cost-effectiveness of hypoallergenic formulas, but these were based on efficacy evidence collected over a maximum of 18 months.^20–24^ A recent publication reported on the incidence of allergic manifestation and cow’s milk tolerance over three years, in children treated with different formulas.^25^ To inform clinical practice and health system efficiency, we compared the cost-effectiveness of amino acid-based formula (AFF), extensively hydrolysed casein formula containing Lactobacillus rhamnosus Gorbach Goldin (EHCF+LGG), extensively hydrolysed whey formula (EHWF), and soy formula (SF), in managing CMPA in children. In this analysis we apply the Consolidated Health Economic Evaluation Reporting Standards approach for reporting economic evaluations.^26^

## METHODS

### Model Structure

We developed a trial-based decision analytic cohort model in Microsoft Excel to simulate the use of hypoallergenic formulas to manage IgE-mediated CMPA in non-breastfed children in the United Kingdom. The model structure was based on a published cost-effectiveness analysis in the United Kingdom,^27^ and applies the UK National Health Service (NHS) perspective for costs. In the United Kingdom, health care is free at the point of access and children are exempt from prescription fees.^28^ Data from a recent trial directly comparing AAF, EHCF+LGG, EHWF, SF, and rice hydrolysed formula were utilized to inform the annual likelihood of allergic manifestations and acquisition of tolerance to cow’s milk protein over the 3-year time horizon of the analysis. A 3-year time horizon was deemed appropriate to assess the cost-effectiveness of the comparators as it covers the period over which infants and children most often present CMPA and its symptoms. Although the model does not assess the impact of CMPA on adult life, we assumed that achieving immune tolerance in early childhood would positively impact future life and would offset the costs of the interventions. We have not included the rice-based formula in the analysis as it is not part of the suitable substitute formulas recommended in the United Kingdom.^9^

**Figure 1.**
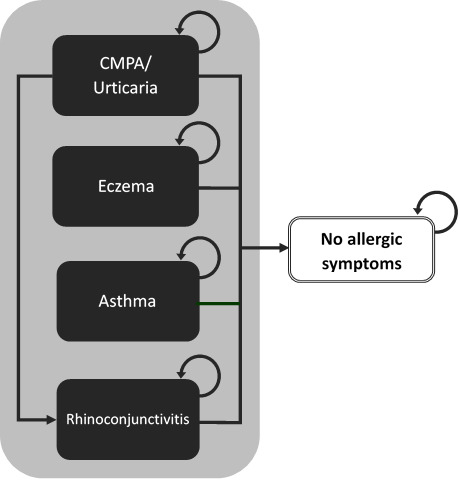
Model Diagram Abbreviations: CMPA, cow’s milk protein allergy.

The model uses a cohort of non-breastfed infants living in the community with IgE-mediated symptoms of CMPA who are at risk of developing allergic manifestations (eczema, asthma, rhinoconjunctivitis, or urticaria) or who can also become symptom-free. Membership in these health states was modelled as mutually exclusive and exhaustive (adding to 1), using annual probabilities of allergic manifestations or being symptom-free reported by Nocerino 2021 and colleagues. Because we had no information on the number of children having multiple symptoms or alternating between symptoms, we accounted only for the incidence of the child’s main allergic manifestation. Average health-care costs of managing CMPA and specific allergic manifestations were attributed to individuals falling into each health state and were aggregated over the 3 years of the model. We assumed that mortality due to CMPA or hypoallergenic formula intake would not be differential between cohorts and have therefore excluded it from the analysis. Figure 1 is a simplified representation of the model structure.

### Allergic Manifestations and Tolerance to Cow’s Milk Protein

The probabilities of allergic manifestations and acquired tolerance to cow’s milk protein were sourced from a 36-month prospective cohort study comparing AAF, EHCF+LGG, EHWF, and SF.^25^ To the best of our knowledge and based on a recently published systematic review,^29^ this is the only trial directly comparing these hypoallergenic formulas and reporting on allergic manifestations and probability of cow’s milk protein tolerance over a 3-year follow-up period. The study recruited 365 non-breastfed infants (73 per comparator) less than 1 year of age and suspected to have IgE-mediated CMPA. At enrolment, all children were symptom-free, had been started on a hypoallergenic milk formula for 15 to 30 days by the clinician referring them to a tertiary specialist centre, and were following a cow’s milk-free diet. IgE-mediated CMPA status was confirmed at baseline using indices of sensitisation (skin prick and specific IgE testing), and by an oral food challenge. At the 12-month follow-ups, children were assessed clinically, and data were collected on the frequency of allergic manifestations, diet status, and compliance to the hypoallergenic formula prescribed.

To check for tolerance to cow’s milk, the researchers also performed an oral food challenge, and a skin prick test to cow’s milk. Parents were asked to bring their children for examination if allergic symptoms occurred, so that the cause of the reaction could be determined. Atopic eczema was diagnosed in children presenting with 3 of the following: pruritus, typical morphology, chronic relapsing, and family history of atopic reactions. Allergic rhinoconjunctivitis was diagnosed based on the presence of rhinitis, nasal congestion, sneezing, itching, rhinorrhoea, or use of rhinitis medication, after having excluded infectious cause. Allergic urticaria was diagnosed after 2 or more occurrences of typical itching eruptions and swelling, after exposure to the same allergen. Asthma was diagnosed in the presence of recurrent wheeze or breathing difficulties and cough that improved with inhaled bronchodilators and steroid therapy, after excluding other possible causes. The primary and secondary outcomes of the study were the incidence of any allergic manifestation and the acquisition of cow’s milk tolerance over the 36-month follow-up, respectively. The authors used a binomial regression model to estimate formula effect on the outcomes of interest having also adjusted for the confounding effect of several variables (gender, duration of breastfeeding 2 months of more, weaning, number of siblings, family risk of allergy, passive smoking, mother smoking status during pregnancy, and exposure to pets). *P*-values below 0.0125 were considered statistically significant. For a more detailed explanation of the study protocol and methodology please refer to the original publication.^25^ At the 3-year timepoint, children receiving EHCF+LGG were statistically significantly less likely to have any allergic manifestation and had a higher probability of being tolerant to cow’s milk. Because Nocerino and colleagues reported the point estimates for the primary and secondary study outcomes for the entire duration of follow-up, the results for years 1 and 2 were extracted from the publication charts using the freely available Engauge Digitizer software. The annual probability of being symptom-free for each comparator was calculated as 1 minus the sum of the probabilities of all the allergic manifestations for that cycle. The efficacy parameters used in the model are shown in Table 1.

**Table 1.** Annual Probabilities of Allergic Manifestations, Being Symptom-free and Tolerant to Cow’s Milk per Comparator

**Formula**	**Time**	**Urticaria**	**Eczema**	**Asthma**	**Rhinoconjunctivitis**	**Symptom-free** ^1^	**CM Tolerance** ^2^
AAF	Year 1	0.151	0.289	0.000	0.178	0.381	0.016
	Year 2	0.097	0.082	0.069	0.138	0.615	0.099
	Year 3	0.041	0.041	0.192	0.041	0.685	0.192
EHCF+LGG	Year 1	0.026	0.000	0.001	0.000	0.972	0.411
	Year 2	0.056	0.096	0.014	0.053	0.782	0.641
	Year 3	0.041	0.041	0.109	0.056	0.753	0.809
EHWF	Year 1	0.081	0.220	0.083	0.082	0.535	0.195
	Year 2	0.055	0.014	0.055	0.069	0.807	0.314
	Year 3	0.083	0.055	0.138	0.152	0.572	0.425
SF	Year 1	0.123	0.247	0.014	0.082	0.534	0.143
	Year 2	0.097	0.054	0.082	0.095	0.671	0.226
	Year 3	0.027	0.069	0.192	0.152	0.559	0.399

^1^Calculated as 1 minus the sum of the annual probabilities of urticaria, eczema, asthma and rhinoconjunctivitis for that cycle.<br>^2^Cumulative probabilities.

Abbreviations: AAF, amino acid-based formula; CM, cow’s milk; EHCF+LGG, extensively hydrolysed casein formula containing Lactobacillus rhamnosus Gorbach Goldin; EHWF, extensively hydrolysed whey formula; SF, soy formula.

### Costs and Resource Use

To estimate the amount of resources required to manage CMPA and its allergic manifestations we used results from a published survey conducted with UK general practitioners reported by Guest and Singh.^27^ We subsequently validated these findings with a UK paediatric allergist to ensure these were still adequate to current UK practice and national guidance. Consequently, we have introduced serum IgE testing as one of the required resources^30^ and have identified the pharmacological formulations suitable for use in paediatrics to include in the costing calculations. We have also assumed all general practitioner (GP) appointments would occur at the surgery, instead of accounting for a proportion of home visits, as these were deemed not to reflect current UK practice as per our clinical expert. At CMPA presentation (year 1 only) the resources used to diagnose and manage the initial gastrointestinal (GI) symptoms of CMPA were imputed to all children. We challenged this assumption in sensitivity analysis as not all children are likely to follow the exact same pathway.^31^ We assumed that the incidence of urticaria symptoms in years 2 and 3 would be due to accidental exposure to cow’s milk or allergic manifestations to other foods (as part of the allergic march) and would be accompanied by GI symptoms. For this reason, resources used to manage GI and urticaria symptoms resulting from the CMPA allergic march were grouped in Table 2.

**Table 2.** Units of Resources Required To Manage CMPA and its Allergic Manifestations*

**Resource**	**Year 1**	**Year 2**	**Year 3**
**Management of CMPA/Urticaria**			
GP visits (mean per patient)	8	3	2
General paediatrician visits (mean per patient)	1	1	1
Paediatric allergist visits (mean per patient)	0.84	0.84	0.84
Dietitian visits (mean per patient)	2.8	1.64	1.64
Accident & emergency attendances (mean per patient)	0.3	0.3	0
Hospital admissions (mean per patient)	0.08	0.05	0.03
Percentage referred to a general paediatrician	50%	0%	0%
Percentage referred to a paediatric allergist	40%	0%	0%
Percentage referred to a dietitian	90%	0%	0%
Percentage prescribed H_2_ antagonists or proton pump inhibitors	28%	0%	0%
Percentage prescribed emollients	85%	0%	0%
Percentage prescribed systemic corticosteroids	3%	3%	3%
Percentage prescribed topical corticosteroids	50%	50%	50%
Percentage prescribed antihistamines	60%	60%	60%
**Management of Eczema**			
GP visits (mean per patient)	4	3	3
General paediatrician visits (mean per patient)	0.9	0.9	0.9
Paediatric dermatologist visits (mean per patient)	0.03	0.06	0.06
Accident & emergency attendances (mean per patient)	0.13	0.13	0.13
Hospital admissions (mean per patient)	0.03	0.03	0.03
Percentage referred to a general paediatrician	45%	0%	0%
Percentage referred to a dermatologist	3%	0%	0%
Percentage prescribed emollients	100%	100%	100%
Percentage prescribed topical corticosteroids	70%	70%	70%
Percentage prescribed antihistamines	50%	50%	50%
**Management of Asthma**			
GP visits (mean per patient)	7	6	5
General paediatrician visits (mean per patient)	2	0.9	0.9
Accident & emergency attendances (mean per patient)	0.2	0.2	0.2
Hospital admissions (mean per patient)	0.4	0.4	0.4
Percentage referred to a general paediatrician	65%	0%	0%
Percentage prescribed systemic corticosteroids	40%	0%	0%
Percentage prescribed Ventolin/beta agonists	100%	100%	100%
Percentage prescribed inhaled corticosteroids	55%	55%	55%
**Management of Rhinoconjunctivitis**			
GP visits (mean per patient)	2	2	2
General paediatrician visits (mean per patient)	0.1	0	0
Accident & emergency attendances (mean per patient)	0.03	0	0
Percentage referred to a general paediatrician	5%	0%	0%
Percentage prescribed antihistamines	80%	80%	80%
**Assumptions on Diagnostic Tests**			
Number of IgE blood tests	2	1	1
Proportion of children	100%	100%	100%
**Mean Number of Prescriptions per Patient**			
Weekly prescription for proton pump inhibitors	3.8	3.8	3.8
Monthly prescription for emollients	3.3	3.3	3.3
Weekly prescription for systemic corticosteroids	0.9	0.9	0.9
Monthly prescription for topical corticosteroids	1.1	1.1	1.1
Monthly prescription for antihistamines	0.3	0.3	0.3
Monthly prescription for inhaled corticosteroids	0.9	0.9	0.9
Monthly prescription of salbutamol 100 mcg/dose	0.9	0.9	0.9

*Guest and Singh 2019

Abbreviations: CMPA, cow’s milk protein allergy; H_2_, histamine H_2_ receptors; IgE, immunoglobulin E.

The estimated costs used to populate the model and its respective sources are shown in Table 3. Appointments and hospital admission costs were sourced from the National Schedule of Costs,^32^ GP and nursing time used values from the Unit Costs of Health and Social Care. We assumed GP appointments would last 9.22 minutes.^33^ The costs for drugs were calculated using primary care tariff prices^34^ weighted according to national dispensing rates,^35^ which were verified by a senior pharmacist. If drug posology was based on body weight, an average value of 12 kg was used for the calculations. Paediatrician appointments were costed as consultant-led non-admitted face-to-face attendance (first appointment WF01A or follow-up WF01B). General paediatrician appointments costs were calculated as a weighted average of paediatric gastroenterology and respiratory medicine (WF01B, WF01A). Allergist and dermatologist appointments used specific costs for paediatric clinical immunology and allergy or dermatology, respectively. Emergency department visits were calculated as the weighted average of all emergency medicine categories. Dietitian appointments were costed as allied health professionals (A03).^32^ When required, costs were inflated to current values using the Pay and Price Index.^33^ Monthly hypoallergenic formula requirements followed the values collected as part of the original survey^27^ with 11 cans being required up to the age of 6 months, 9 cans for children aged 6 to 12 months, 7 cans from 12 to 18 months, and 6 cans from 18 to 24 months. The average prices for each comparator were calculated using values sourced from the NHS Dictionary of Medicines and Devices,^36^ which were weighted according to the market share of formulations available for each type of hypoallergenic formula.^35^

**Table 3.** Costs of Resources Used in the Economic Model

**Resources**	**UK prices**	**Source**
General practitioner clinic visit	£33.19	NHS 2020a
General practitioner home visit	£0.00	
Initial visit general paediatrician	£271.96	
Follow-up visit general paediatrician	£206.02	
Initial visit paediatric allergist	£241.31	
Follow-up visit paediatric allergist	£211.86	
Initial visit paediatric dermatologist	£159.47	
Follow-up visit paediatric dermatologist	£132.58	
Initial visit dietitian	£89.90	
Follow-up visit dietitian	£89.90	
Accident and emergency attendance ^₸^	£166.05	
Hospital admission ^¥^	£1017.14	
Weekly prescription for proton pump inhibitors	£1.68	NHS 2020b, NHS 2020c*
Monthly prescription for emollients	£30.87	
Weekly prescription for systemic corticosteroids	£2.29	
Monthly prescription for topical corticosteroids	£8.43	
Monthly prescription for antihistamines	£2.19	
Monthly prescription for inhaled corticosteroids	£14.55	
Monthly prescription of salbutamol 100 mcg/dose	£11.78	
AAF (400 g can)	£23.61	NHS 2021, NHS 2020c
EHCF+LGG (400 g can)	£11.21	
EHWF (800 g can)	£19.26	
SF (800 g can)	£10.67	
IgE blood tests ^§^	£21.45	NICE 2021

* NHS Drug Tariff costs weighted using PCA data, July 2020.

± Weighted average of Emergency Medicine categories (T01A, T01NA, T02A, T02NA, T03A, T03NA, T04A, T04NA).

¥ Weighted average of elective and non-elective short and long stay (PD12A-C, PF25A-E, PF26A-C, PF28A-E, PJ35A-D, PJ66A-C and PX50A-C).

§ Not available in the National Schedule of reference cost. Micro costing approach was taken using price for 1 allergy tested as reported in NICE CG116.^30^

Abbreviations: AAF, amino acid-based formula; CM, cow’s milk; EHCF+LGG, extensively hydrolysed casein formula containing Lactobacillus rhamnosus Gorbach Goldin; EHWF, extensively hydrolysed whey formula; IgE, immunoglobulin E; mcg, micrograms; SF, soy formula.

### Measures of Health Gain

Due to the difficulties in assessing utilities in infancy and early childhood,^37^ model results were reported as cost per child tolerant to cow’s milk at 3 years and cost per child free from allergic symptoms of CMPA at 3 years. The probability of being free from allergic manifestations was calculated as the inverse of the probability of having any allergic manifestation at the end of the 3 years of the study. The probability of being cow’s milk tolerant used the estimates reported by Nocerino 2021.^25^

### Model Results

The incremental costs-effectiveness ratios (ICERs) were calculated as the incremental costs divided by the incremental probabilities of being free from allergic manifestations or being cow’s milk tolerant at the end of the model time horizon. Additionally, we reported the cost per life-years lived without allergic manifestations and life-years lived with cow’s milk tolerance. We discounted costs and health consequences at a 3.5% rate in years 2 and 3 of the analysis.^38^ Despite the absence of a formal willingness to pay (WTP) threshold for the health effects in the model, we report the net monetary benefit for each strategy, allowing for a more intuitive ranking of strategies according to their cost-effectiveness. We have used the minimum and maximum cost per health benefit across strategies to become the low and high WTP thresholds, respectively. The resulting thresholds set the WTP for an additional unit of benefit to be identical to the estimated total cost of the cheapest or the most expensive strategies, respectively. This analysis essentially sets the range of opportunity costs of not using the most cost-effective strategy.

### Sensitivity Analyses

As part of the deterministic sensitivity analyses, we ran 2 scenarios in addition to the base case. Firstly, we reduced the proportion of children presenting with GI symptoms of CMPA in year 1 to 60%.^24,25,39^ As a second scenario, we varied health-care utilization by 30% to reflect country-wide heterogeneity.

One-way sensitivity analyses were conducted using the lower and upper bounds of the 95% confidence intervals of all deterministic inputs to examine the impact of the most influential parameters on the results of the model. These findings were summarized in a tornado diagram.

A Monte Carlo simulation was used to account for parameter uncertainty by sampling 1000 times from distributions assigned to model inputs. Annual probabilities of allergic manifestations and being symptom-free were sampled from Dirichlet distributions using events of interest and complements reported by Nocerino and colleagues. Annual probabilities of being cow’s milk-tolerant for the different comparators were sampled from beta distribution also by using frequency of the event of interest and complement.^40^ Because we did not have a measure of variance for published costs, we assigned cost inputs to uniform distributions and varied mean estimates by 40%.

## RESULTS

### Base Case

In the base case, children receiving EHCF+LGG were associated with a higher probability of being symptom-free at the 3-year time-horizon and with lower total costs (dominant), compared to children on SF, EHWF, or AAF. At the set WTP thresholds, EHCF+LGG had the highest net monetary benefit as the most cost-effective strategy at its set price. Similar results were obtained using the probability of being tolerant to cow’s milk 3 years after introduction of hypoallergenic formula. Children receiving EHCF+LGG accounted for lower total costs and had a higher probability of being tolerant to cow’s milk protein, compared to children on the alternative comparators. EHCF+LGG had the highest net monetary benefit at a WTP of £2462 and £25 088 per additional child tolerant to cow’s milk. When using cumulative life years without symptoms and life years tolerant to cow’s milk protein at 3 years as the denominators to the ICERs, the conclusions of the model did not change, with EHCF+LGG being the most cost-effective strategy. The deterministic results of the model are shown in Table 4. Because extensively hydrolysed formulas are the first line recommendation for children with CMPA in the UK,^9^ EHCF+LGG, and EHWF were compared head-to-head. Over a 3-year period, children receiving EHCF+LGG used £476 less health-care resources, had a 0.181 higher probability of being symptom-free, and a 0.358 higher probability of tolerance to cow’s milk protein. In other words, 3 years after CMPA presentation, approximately 8 out of 10 children receiving EHCF+LGG would be symptom-free, compared to 6 out of 10 children in the EHWF group. Over the same period, approximately 8 out of every 10 children receiving EHCF+LGG would have acquired immune tolerance to cow’s milk whilst this would be true in only 4 of 10 children on EHWF.

**Table 4.** Base Case Deterministic Results per Cow’s Milk Tolerant or Symptom-free Child (discounted)

**Comparator**	**Total costs**	**Effects**	**Incremental costs**	**Incremental effects**	ICER^*^	**Net Monetary Benefit**	
**Probability of being symptom free at 3 years**						**WTP £2645**	**WTP £7046**
EHCF+LGG	£1859	0.703				£0	£3093
SF	£1978	0.522	£119	-0.181	dominated	-£598	£1698
EHWF	£2335	0.534	£476	-0.169	dominated	-£923	£1427
AAF	£4505	0.639	£2645	-0.064	dominated	-£2814	£0
**Life years without symptoms at 3 years**						**WTP £765**	**WTP £2709**
EHCF+LGG	£1859	2.431				£0	£4922
SF	£1978	1.704	£119	-0.727	dominated	-£675	£2776
EHWF	£2335	1.725	£476	-0.705	dominated	-£1016	£2478
AAF	£4505	1.615	£2645	-0.816	dominated	-£3270	£0
**Probability of cow’s milk tolerance at 3 years**						**WTP £2462**	**WTP £25 088**
EHCF+LGG	£1859	0.755				£0	£17 085
SF	£1978	0.373	£119	-0.383	dominated	-£1061	£7367
EHWF	£2335	0.397	£476	-0.358	dominated	-£1358	£7618
AAF	£4505	0.180	£2645	-0.576	dominated	-£4062	£0
**Life years with cow’s milk tolerance at 3 years**						**WTP £1 042**	**WTP £15 480**
EHCF+LGG	£1859	1.785				£0	£25 766
SF	£1978	0.734	£119	-1.051	dominated	-£1214	£9376
EHWF	£4505	0.895	£476	-0.889	dominated	-£1403	£11 522
AAF	£2954	0.291	£2645	-1.494	dominated	-£4201	£0

* Calculated as the difference in costs divided by the difference in effects.

Abbreviations: AAF, amino acid-based formula; EHCF+LGG, extensively hydrolysed casein formula containing Lactobacillus rhamnosus Gorbach Goldin; EHWF, extensively hydrolysed whey formula; ICER, incremental cost-effectiveness ratio; SF, soy formula; WTP, willingness to pay threshold.

**Figure 2.**
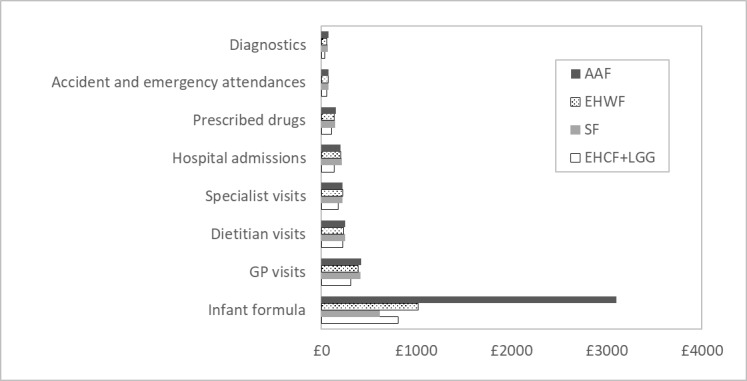
Resource Consumption in the Cost-effectiveness Model Abbreviations: AAF, amino acid-based formula; EHCF+LGG, extensively hydrolysed casein formula containing Lactobacillus rhamnosus Gorbach Goldin; EHWF, extensively hydrolysed whey formula; SF, soy formula.

Resource consumption by health-care categories in the model is represented in Figure 2. Infant formula accounted for the largest proportion of total costs averaging 47% across comparators, with a minimum of 31% for SF and a maximum of 69% for AAF. Visits to GPs constituted the second highest cost component, approximately 16% of total costs across comparators. Dietitian visits, specialist appointments, and hospital admissions represented 8% to 10% of total costs, whilst medicines, emergency department attendances, and diagnostics corresponded to a maximum of 5% of total cost amongst all comparators. If excluding infant formula costs, our analysis predicted that children receiving EHCF+LGG incurred 20%, 23%, and 25% less health-care resources compared to those on EHWF, SF, and AAF, respectively. Differences in costs were dependent on the incidence of allergic manifestations between comparators and, consequently, a differential use of health-care resources.

### Sensitivity Analysis

#### Deterministic Sensitivity Analysis

Changing the proportion of children presenting with GI symptoms of CMPA in year 1 to 60% had no material impact on the model conclusions, with EHCF+LGG still being the dominating strategy for both model outcomes. Reducing the incidence of GI symptoms led to an indirect reduction of health-care resource consumption, which caused clinical nutrition to account for 56% of total costs across strategies. In the second scenario, we increased and decreased health-care utilization by 30% to reflect local variation in clinical practice. As before, the conclusions of the analysis did not change with EHCF+LGG being the most cost-effective strategy.

#### One-way Sensitivity Analysis

**Figure 3.**
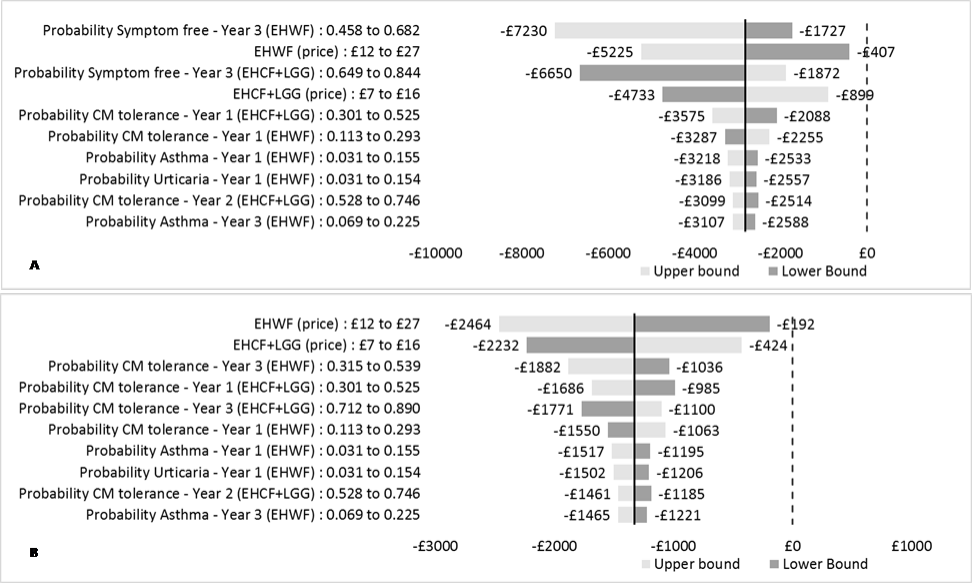
Tornado Diagrams for EHCF+LGG Versus EHWF A: Tornado diagram for the probability of being symptom free at 3 years; B: Tornado diagram for the probability of being tolerant to cow’s milk at 3 years. Abbreviations: CM, cow’s milk; EHCF+LGG, extensively hydrolysed casein formula containing Lactobacillus rhamnosus Gorbach Goldin (EHCF+LGG); EHWF, extensively hydrolysed whey formula. The tornado diagram represents how varying inputs between specific ranges influences the outcomes. The bold vertical line represents the base case results. The horizontal bars are stacked in order of decreasing width, with more influential inputs at the top. Horizontal lines crossing the vertical dashed line would represent a change in the conclusions of the model caused by varying a single parameter.

Each tornado diagram depicts the variation around the ICER resulting from changing the 10 most influential parameters to the lower and upper bounds of their 95% confidence interval. ICERs take negative values because EHCF+LGG dominates the remaining comparators. When the model was run for the probability of being symptom-free outcome, model results were most sensitive to the probability of being symptom-free at year 3 in children receiving EHWF and EHCF+LGG, and to formula price. Varying these parameters in the one-way analysis did not influence the model conclusions. Similarly, formula price and the probability of tolerance to cow’s milk at 1 and 3 years were the most influential parameters when tolerance to cow’s milk was the main outcome of the analysis. The results were once more robust to parameter variation with none of the inputs causing the ICER to point to a different conclusion of the analysis. The results of the one-way sensitivity analyses comparing EHCF+LGG with EHWF are shown in Figure 3. Additional results of one-way sensitivity analyses are available in the **Supplemental Material**.

#### Probabilistic Sensitivity Analysis

**Figure 4.**
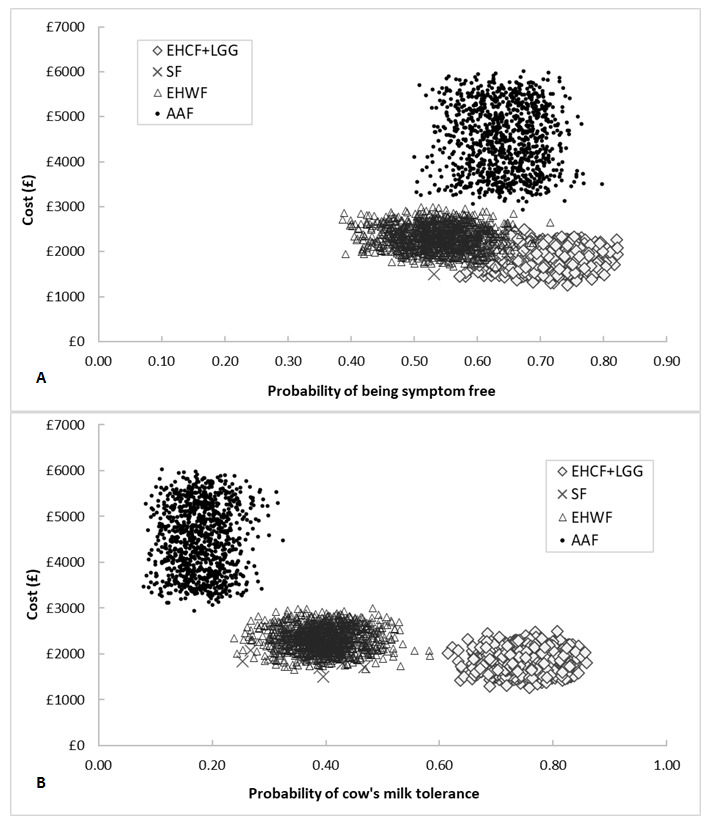
Cost-effectiveness Planes for Base Case Probabilistic Results A: cost-effectiveness plane showing probabilistic results for the probability of being symptom-free at 3 years; B: cost-effectiveness plane showing probabilistic results for the probability of being tolerant to cow’s milk at 3 years. Abbreviations: AAF, amino acid-based formula; EHCF+LGG, extensively hydrolysed casein formula containing Lactobacillus rhamnosus Gorbach Goldin (EHCF+LGG); EHWF, extensively hydrolysed whey formula.

**Figure 5.**
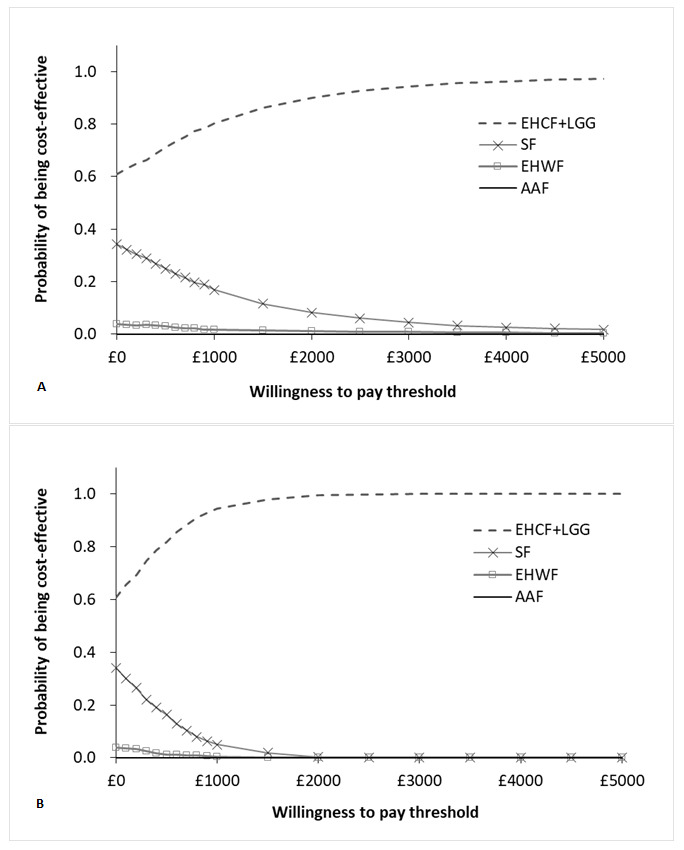
Cost-effectiveness Acceptability Curves for Base Case Probabilistic Results A: cost-effectiveness acceptability curve for the probability of being symptom-free at 3 years; B: cost-effectiveness acceptability curve for the probability of being tolerant to cow’s milk at 3 years. Abbreviations: AAF, amino acid-based formula; EHCF+LGG, extensively hydrolysed casein formula containing Lactobacillus rhamnosus Gorbach Goldin (EHCF+LGG); EHWF, extensively hydrolysed whey formula.

The results of 1000 iterations of the probabilistic sensitivity analysis were almost identical to the deterministic results. The results of the probabilistic sampling are plotted in the cost-effectiveness plane depicted in Figure 4. For tabled average results, please refer to the **Supplemental Material**. For WTP values of £2650 and £7045 per child free from allergic manifestations at 3 years, our model predicted that EHCF+LGG had a 93% and a 98% probability of being the most cost-effective strategy, respectively. For willingness to pay of £2461 and £25 039 per cow’s milk tolerant child at 3 years the probability of EHCF+LGG being cost-effective was 100%. The cost-effectiveness acceptability curves produced for both model outcomes are shown in Figure 5. The probabilistic result for the additional outcomes in the model were similar to those described above and are described in more detail in the **Supplemental Material**.

## DISCUSSION

Breastfeeding is widely recognised as the preferred form of nutrition for new-born children.^12,41,42^ Nonetheless, it can be challenging to completely exclude dairy, and potentially soy,^9^ from the diet of mothers choosing to breastfeed their children. This can lead to incomplete symptom resolution in children with CMPA.^2^ Additionally, mothers may choose to give up breastfeeding due to difficulties in latching on, perceived lack of satisfaction with breast milk alone or due to other lactation or nutrition issues.^43^ Consequently, hypoallergenic formulas play an important role in replacing or supplementing maternal milk in children who are allergic to cow’s milk. It is, therefore, crucial that hypoallergenic formula choice is guided by strict efficacy and cost-effectiveness criteria.

Based on previously reported investigations of allergic manifestations and CMPA tolerance, our modelled evaluation reveals that children receiving EHCF+LGG required less NHS resources such as medical appointments, hospital admissions, and medicines, compared to alternative milk formula substitutions. Model exploration in sensitivity analyses has shown that our results are robust to new assumptions and input variability. We used peer reviewed efficacy evidence directly comparing the formulas relevant to UK clinical practice.^25^ Resource estimation was obtained from GPs practicing in the United Kingdom and has been used in a previously published cost-effectiveness analysis.^27^ The prices of resources were obtained from standard, publicly available UK sources.

### Limitations

There are some limitations to our economic analysis that are worth considering in relation to interpreting the results described. Firstly, the model is based on data from a non-randomised study conducted in a single European country. Generalizing the results of the trial underpins the assumption that children living with CMPA in the United Kingdom have allergic manifestations and responses to hypoallergenic formulas similar to those enrolled in the original study. Study arms were well-balanced, and the study was powered to detect a difference in the overall incidence of allergic manifestations between cohorts treated with hypoallergenic formulas—one of the main outcomes of the model. Because the probability of tolerance to cow’s milk allergy was a secondary outcome, the study may not have been powered to detect a difference between comparators. However, study results are in line with previous evidence of the effect of hypoallergenic formulas on the incidence of allergic manifestations^44–47^ and acquisition of immune tolerance^24,29,39,48–50^ and were, therefore, deemed appropriate to inform treatment efficacy. Additionally, to the best of our knowledge, there is no randomized study comparing the formula products relevant for the UK analysis.^29^

One possible consequence of not randomizing children to hypoallergenic formulas is the fact that the cohorts could substantially differ from each other, which would introduce bias in the analysis. There were no obvious differences in the demographic characteristics between cohorts reported by Nocerino et al. and the authors have adjusted for confounding using binary regression. We can hypothesise that children receiving AAF were more likely to have severe CMPA (not at random), as this type of formula is often prescribed to children with more severe atopic manifestations or atopic backgrounds.^9^ Given the higher acquisition cost of AAF, and the results of our analysis, it seems unlikely that AAF would be the most cost-effective first line option in the management of CMPA in the United Kingdom, which, in theory, would undermine the bias effect associated with this subpopulation with more severe disease. However, assessing the cost-effectiveness of second line treatment of CMPA goes beyond the scope of this research.

The data informing health-care utilisation and symptom management in children presenting with CMAP in the United Kingdom was obtained from a survey conducted among 4 GPs, which is a very small sample. To the best of our knowledge, there is no alternative publication to inform these inputs in the model and there may be nationwide variability in the way children with CMPA are managed. For example, paediatric allergists may not be equally available in urban and rural areas, admission to hospital may be influenced by the availability of out-of-hours health-care provision and some clinicians may be more inclined to prescribe certain medicines than others. We have challenged the face validity of the utilized inputs by subjecting them to scrutiny by an experienced UK clinician. In addition, we have varied total health-care costs in sensitivity analysis, which has not produced changes to the model conclusions. Utilizing a more representative survey of resource utilization would most likely affect the absolute value of resource utilization, but *a priori* this would not be differential between formulas and would, therefore, not affect our conclusions, as reflected in sensitivity analysis. EHCF+LGG has been associated with a faster improvement in CMPA symptoms and cow’s milk tolerance, leading to a faster reduction in health-care needs and formula utilization.

The time horizon of the model was limited to 36 months, mimicking the follow-up duration of the clinical trial. We have not attempted to extrapolate the effect of hypoallergenic formulas, as this was thought to increase uncertainty in the analysis, and because it did not modify the conclusions of a previously published cost-effectiveness analysis.^27^

The outcome measures of interest assessed in our cost-effectiveness analysis were the likelihood of being free from allergic symptoms and cow’s milk tolerance, rather than quality-adjusted life years, commonly performed in UK economic evaluations.^38^ There are several reasons for not including quality-adjusted life years in this cohort of children that are worth considering. Firstly, capturing utility parameters in children below the age of 5 is not free from methodological challenges.^37^ Secondly, the frequency, intensity and duration of each allergic manifestation varies greatly between children, which would impact the accuracy of the quality of life estimates. Finally, symptoms of CMPA and associated allergic manifestations are distressing for children but can also impact the well-being of families. Due to the uncertainty in quality-adjusted life years estimation and broader consequences of CMPA symptoms, we perceived the absence of allergic manifestations and cow’s milk tolerance as relevant outcomes, meaningful to both the clinical community and families.

Our model does not consider adverse events associated with formula intake, which is in line with the study informing the efficacy parameters.^25^ We assumed that infants were established on clinical nutrition, not having modelled the costs and consequences of allergic reactions to hypoallergenic formulas and its management. In future research, it could be relevant to model the most cost-effective sequence to introduce clinical nutrition in non-breastfed children with CMPA.

Finally, the model is likely to underestimate the true burden of CMPA. We do not account for the impact of CMPA on child development, concomitant health problems, and long-term consequences of the allergic march. Similarly, we do not include the externalities falling on parents and families that come as consequence of CMPA-related disruption to sleeping patterns, family nutrition, and well-being more generally.

## CONCLUSION

Achieving efficiency in health-care delivery is a goal that benefits everyone. Achieving this goal requires the application of timely economic evaluation to consider the full range of outcomes and costs in relation to different interventions. The investigation reported here demonstrates the benefits of oral tolerance to cow’s milk and reduction of allergic manifestations when treating CMPA with EHCF+LGG based on a recently published investigation. Immune tolerance is likely to positively affect child development, families’ well-being, and substantially reduce the costs of health care and infant formula. Despite limitations in the data, the analysis suggests that EHCF+LGG is the most cost-effective strategy to manage non-breastfed children with a diagnosis of CMPA in the United Kingdom.

## Supplementary Material

Online Supplementary Material
