# Author Correction: Cost-effectiveness Analysis of Hypoallergenic Milk Formulas for the Management of Cow’s Milk Protein Allergy in the United Kingdom

**DOI:** 10.36469/001c.28124

**Published:** 2021-10-06

**Authors:** Rui Martins, Mark P. Connolly, Eleanor Minshall

**Affiliations:** 1 Global Market Access Solutions, Health Economics Unit, St-Prex, Switzerland; 2 Unit of PharmacoEpidemiology & PharmacoEconomics, Department of Pharmacy, University of Groningen, Groningen, The Netherlands; GMAS Services LTD, London, England; 3 Sheffield Children’s NHS Foundation Trust, Sheffield, United Kingdom

**Keywords:** cost-effectiveness, atopic march, hypoallergenic formula milk, cow’s milk protein allergy

Correction to: https://doi.org/10.36469/jheor.2021.26010

Published online: Aug 6, 2021

We have identified an error in the cost of one of the comparators under evaluation which, despite not changing the conclusions of the cost-effectiveness analysis impacted some of its results. In the original publication we estimated treatment costs of amino acid-based formula (AAF) based on a price per 800 g tin, whilst the actual size of the container is only 400 g. In effect, the utilized price was half of what it should be. We have corrected this input and updated the sections of the manuscript and supplemental data file where the cost of AAF treatment was mentioned. As a consequence, based on changes to the cost of treatment with AAF, results from the deterministic and probabilistic analyses have changed. In conclusion, AAF remained dominated but its absolute and incremental costs increased. The original article has been updated online.

