# Supplementary material for: Cost-effectiveness Analysis of Hypoallergenic Milk Formulas for the Management of Cow’s Milk Protein Allergy in the United Kingdom
Source: J Health Econ Outcomes Res. 2021 Aug 6;8(2):14–25. doi: 10.36469/jheor.2021.26010 (PMC8346363; doi:10.36469/jheor.2021.26010)
Supplement: Online Supplementary Material [file jheor_2021_8_2_26010_72916.pdf]

Journal of Health Economics and Outcomes Research

Supplementary Online Material

Martins R, Connolly MP, Minshall E. Cost-effectiveness analysis of hypoallergenic milk formulas for the management of cow's milk protein allergy in the United Kingdom. *JHEOR*. 2021;8(2)14-25.
doi:10.36469/jheor.2021.26010

Base Case Probabilistic Results
Table S1. Base Case Probabilistic Results per Cow's Milk Tolerant of Symptom Free Child
Additional Results of the One-way Sensitivity Analyses
Figure S1. Tornado Diagrams for EHCF+LGG versus EHWF
Figure S2. Cost-effectiveness Planes
Figure S3. Cost-effectiveness Acceptability Curve for Additional Base Case Probabilistic Results at the 3-year Time Horizon

This supplementary material has been provided by the authors to give readers additional information about their work.

This is an open-access article distributed under the terms of the Creative Commons Attribution 4.0 International License (CCBY-4.0). View this license's legal deed at http://creativecommons.org/licenses/by/4.0 and legal code at http://creativecommons.org/licenses/by/4.0/legalcode for more information.

Base Case Probabilistic Results

Table S1. Base Case Probabilistic Results per Cow's Milk Tolerant of Symptom Free Child

Comparator	Total Costs	Effects	Incremental Costs	Incremental Effects	ICER*	Net Monetary Benefit	
**Probability of Being Symptom Free at 3 Years**						**WTP £2650**	**WTP £7045**
EHCF+LGG	£1860	0.702				£0	£3084
SF	£1973	0.524	£113	-0.178	dominated	-£584	£1719
EHWF	£2341	0.536	£481	-0.166	dominated	-£920	£1435
AAF	£4511	0.640	£2651	-0.061	dominated	-£2814	£0
**Life Years without Symptoms at 3 Years**						WTP £765	WTP £2798
EHCF+LGG	£1860	2.432				£0	£4945
SF	£1973	1.706	£113	-0.726	dominated	-£668	£2800
EHWF	£2341	1.733	£481	-0.698	dominated	-£1015	£2509
AAF	£4511	1.612	£2651	-0.820	dominated	-£3278	£0
**Probability of Cow's Milk Tolerance (3 years)**						WTP £2461	WTP £25 039
EHCF+LGG	£1860	0.756				£0	£17 061
SF	£1973	0.372	£113	-0.383	dominated	-£1057	£7346
EHWF	£2341	0.397	£481	-0.359	dominated	-£1364	£7594
AAF	£4511	0.180	£2651	-0.575	dominated	-£4067	£0
**Life Years with Cow's Milk Tolerance at 3 Years**						WTP £1040	WTP £15 456
EHCF+LGG	£1860	1.787				£0	£25 764
SF	£1973	0.732	£113	-1.055	dominated	-£1211	£9338
EHWF	£2341	0.897	£481	-0.890	dominated	-£1408	£11 521
AAF	£4511	0.292	£2651	-1.495	dominated	-£4207	£0

* Calculated as the difference in costs divided by the difference in effects.

Abbreviations: AAF, amino acid-based formula; EHCF+LGG, extensively hydrolysed casein formula containing Lactobacillus rhamnosus Gorbach Goldin; EHWF, extensively hydrolysed whey formula; ICER, incremental cost-effectiveness ratio; SF, soy formula; WTP, willingness to pay threshold.

When considering life years without symptoms at the 3-year time horizon as the main outcome in the model, EHCF + LGG had 97% and 100% probabilities of being the most cost-effective strategy at the willingness to pay threshold of £765 and £2798, respectively.

When life years of being tolerant to cow's milk were considered, EHCF + LGG was associated with 100% probability of being the most cost-effective strategy at the £1040 and £15 456 willingness to pay thresholds.

Figure S1. Tornado Diagrams for EHCF+LGG versus EHWF

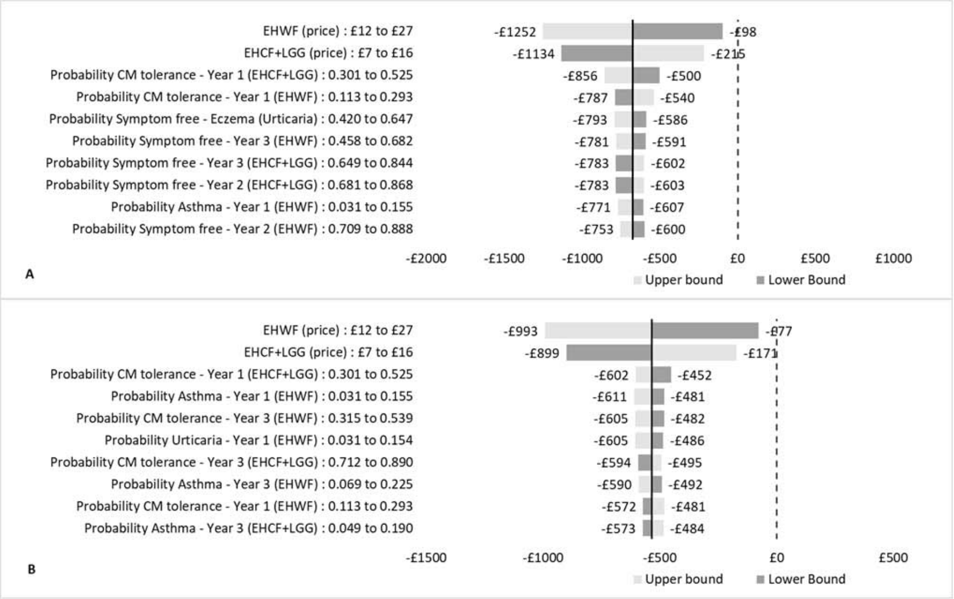

A: Tornado diagram for life years of being symptom free at the 3-year time horizon; B: Tornado diagram for life years being tolerant to cow's milk at the 3-year time horizon.

Abbreviations: CM, cow's milk; EHCF+LGG, extensively hydrolysed casein formula containing Lactobacillus rhamnosus Gorbach Goldin (EHCF+LGG), EHWF, extensively hydrolysed whey formula. The tornado diagram represents how varying inputs between specific ranges influences the outcomes. The bold vertical line represents the base case results. The horizontal bars are stacked in order of decreasing width, with more influential inputs at the top. Horizontal lines crossing the vertical dashed line would represent a change in the conclusions of the model caused by varying a single parameter.

Figure S2. Cost-effectiveness Planes

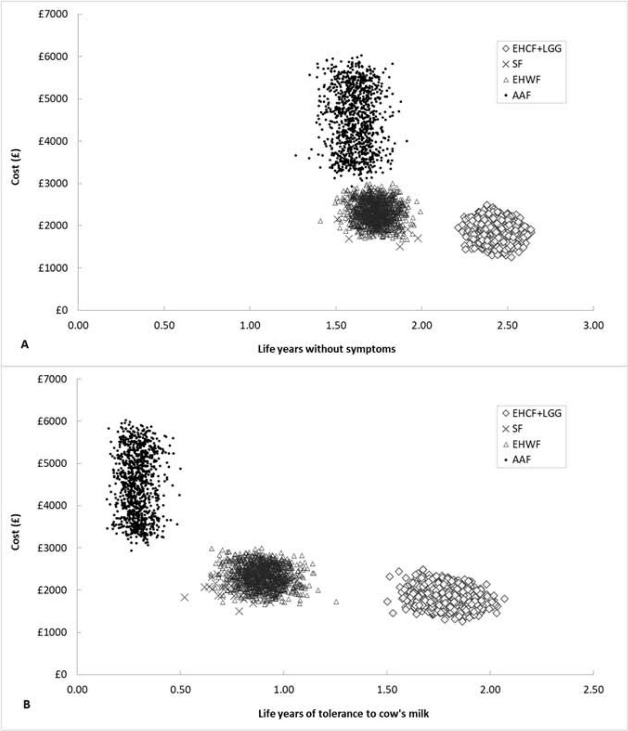

A: Cost-effectiveness plane for life years of being symptom-free at the 3-year time horizon; B: cost-effectiveness plane for life years of being tolerant to cow's milk at the 3-year time horizon.

Abbreviations: AAF, amino acid-based formula; EHCF+LGG, extensively hydrolysed casein formula containing Lactobacillus rhamnosus Gorbach Goldin (EHCF+LGG); EHWF, extensively hydrolysed whey formula; SF, soy formula.

Figure S3. Cost-effectiveness Acceptability Curves

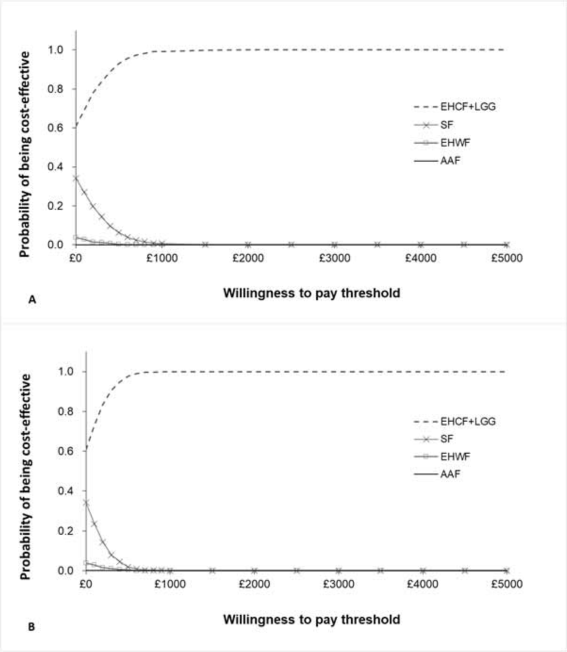

A: Cost-effectiveness acceptability curve for the life years of being symptom-free at the 3-year time horizon; B: cost-effectiveness acceptability curve for life years of being tolerant to cow's milk at the 3-year time horizon.

Abbreviations: AAF, amino acid-based formula; EHCF+LGG, extensively hydrolysed casein formula containing Lactobacillus rhamnosus Gorbach Goldin (EHCF+LGG); EHWF, extensively hydrolysed whey formula; SF, soy formula.
